# Efficacy of EA575 as an Antitussive and Mucoactive Agent in Preclinical In Vivo Models

**DOI:** 10.3390/biomedicines13071673

**Published:** 2025-07-08

**Authors:** Matthias Hufnagel, André Rademaekers, Anika Weisert, Hanns Häberlein, Sebastian Franken

**Affiliations:** 1 Engelhard Arzneimittel GmbH & Co. KG, 61138 Niederdorfelden, Germany; 2 Institute of Biochemistry and Molecular Biology, Medical Faculty, University of Bonn, 53115 Bonn, Germany; 3 Department of Natural Sciences, Institute for Functional Gene Analytics, Bonn-Rhein-Sieg University of Applied Sciences, 53359 Rheinbach, Germany

**Keywords:** ivy leaf dry extract, EA575, antitussive, respiratory disease, cough, mucoactive agent

## Abstract

**Background:** The efficacy of EA575 in the treatment of respiratory diseases is described in various clinical studies, improving patients’ disease-related symptoms. However, mechanistic in vivo data proving its beneficial effects are limited. **Methods:** Focusing on the treatment of acute airway inflammation and accompanying cough, this study aimed to elucidate antitussive and mucoactive properties of EA575, applying two animal models. Animals were treated orally twice daily for 7 days, resulting in 43, 215.2, or 430.5 mg/kg bw/d of EA575. Antitussive effects were investigated within an acute lung inflammation model of bleomycin-treated guinea pigs after citric acid exposure. Hereby, the number of coughs, enhanced pause (penH), and bronchoalveolar lavage fluid (BALF) were investigated. Mucoactivity of EA575 was assessed within a murine model, determining phenol red concentration in BALF. **Results:** EA575 treatment within the acute lung inflammation model reduced cough events up to 56% while reducing inflammatory cell influx in BALF dose-dependently, e.g., reducing neutrophils in BALF up to 70.9%. This suggests a strong connection between anti-inflammatory and antitussive properties of EA575. Furthermore, penH decreased in a dose-dependent manner, suggesting an ease in respiration. Mucoactivity was shown by a dose-dependent increase in phenol red concentration in BALF up to 38.9%. Notably, EA575/salbutamol co-administration resulted in enhanced phenol red secretion compared to respective single administrations. **Conclusions:** These data highlight the benefits of EA575 in treating cough-related respiratory diseases, particularly when accompanied by sputum, as EA575 has been shown to obtain mucoactivity. Furthermore, the combinatory effect of EA575/salbutamol treatment provides a foundation for future research in the treatment of chronic respiratory diseases.

## 1. Introduction

*Hedera helix* (ivy) preparations are widely used within the symptomatic treatment of inflammatory-related respiratory diseases. The efficacy and safety of specific dry, liquid, and soft extracts are well described within the European Medicines Agency (EMA) Committee on Herbal Medicinal Products (HMPC) assessment, which states the beneficial use of ivy leaf extract preparations as an expectorant in cases of productive cough [1]. EA575 represents a unique ivy leaf dry extract (DER 5–7.5:1, extraction solvent: 30% ethanol), whose clinical efficacy and safety in cough-related diseases, such as acute bronchitis, have been shown in numerous studies, including placebo-controlled clinical trials and non-interventional studies [2,3]. EA575-containing medicinal products are recommended for the treatment of acute inflammation-related airway diseases accompanied by cough and chronic inflammatory airway diseases [4]. The composition of EA575 has been the subject of extensive research and includes several saponins, flavonoids, and phenolic acids, such as α-/β-hederin, hederacoside B, C, and D, rutin, kaempferol-3-O-rutinoside, and dicaffeoylquinic acids, among others [5]. The anti-inflammatory properties of EA575 have been studied extensively in vitro. In detail, EA575 ameliorated the IL-6 secretion of stimulated murine macrophages J774.2 [6] and Calu-3 cells [7]. Additionally, it was shown recently that EA575 reduces the secretion of proinflammatory cytokines in immature human monocyte-derived dendritic cells [8]. Moreover, the deactivation of the NF-κB pathway seems to play a crucial role in the anti-inflammatory response triggered by EA575. In a high-level overview, TNF-α-induced NF-κB transcriptional activity was reduced by EA575 in HEK cells [9,10], as well as in THP-1 and A549 cells [10]. Mechanistically, it is postulated that a specificity switch of IKKβ, with subsequent reduction in nuclear NF-κB translocation, results from EA575 treatment [10]. Besides its anti-inflammatory properties, EA575 is known to modulate different receptors, e.g., β_2_ adrenergic and adenosine receptor A_2B_. In detail, increased cAMP resulting from indirect β_2_ adrenergic receptor activation and increased receptor binding in stimulated HEK293-β_2_AR-SNAP cells after EA575 treatment was described [11]. Moreover, the inhibition of β-arrestin 2 recruitment and subsequent prolonged β_2_ adrenergic receptor activation caused by EA575 incubation was observed in HEK cells. Additionally, biased β_2_-adrenegic receptor signaling of EA575 was elucidated, increasing the G-protein/cAMP pathway while decreasing GRK2/β-arrestin 2 signaling [9]. These observations are the molecular basis for the secretolytic and bronchospasmolytic effects of EA575, since increased cAMP levels and changes in cellular Ca^2+^ homeostasis occur upon β_2_ adrenergic receptor activation [12,13,14]. In contrast to the ability of EA575 to activate β_2_ adrenergic receptor, adenosine receptor A_2B_ inhibition by EA575 is indicated via attenuation of respective pathway response in the form of decreased cAMP levels and β-arrestin 2 recruitment in HEK293 reporter cell lines. The decreased intracellular cAMP concentration resulted in inhibition of cAMP response elements (CRE) [7]. However, in vivo data on the efficacy of EA575 are scarce. Therefore, this study aims to elucidate the inflammation-related antitussive efficacy of ivy leaf dry extract EA575, as well as its mucoactivity. Both parameters represent symptoms of acute airway inflammation, such as acute bronchitis; thus, the non-clinical efficacy of EA575 for the treatment of respective diseases is investigated.

## 2. Materials and Methods

### 2.1. Materials

Bleomycin (cat. no. B1141000), dexamethasone (cat. no. D2915), citric acid (cat. no. 27109), Tween-80 (cat. no. P1754), phenol red (cat. no.114529), and sodium hydroxide (cat. no. 28-3040) were purchased from MiliporeSigma (Merck KGaA, Darmstadt, Germany). Phosphate-buffered saline (cat. no. 10010023) was obtained from Thermo Fischer Scientific (Waltham, MA, USA), and salbutamol (cat. no. 0634) was purchased from Tocris Biosciences (Bristol, UK). Pentobarbitone (cat. no. XVD132) was supplied by Ecuphar (Greifswald, Germany), and isoflurane (cat. no. FDG9623) was provided by Baxter Healthcare (Deerfield, IL, USA).

### 2.2. Test Item

Ivy leaf dry extract EA575 is well-characterized, consisting of various constituents from the substance classes of flavonoids, saponins, and phenolic acids identified via LC-MS analysis [5]. The DER of this extract is 5–7.5:1 (solvent: 30% ethanol). The test item (batch number 21N0160) used within this study was analyzed according to the method described by Greunke and colleagues, observing a corresponding fingerprint. The following compounds were identified: protocatechuic acid, neochlorogenic acid, chlorogenic acid, cryptochlorogenic acid, rutin, kaempferol-3-O-rutinoside, 3, 4-, 3, 5-, and 4, 5 dicaffeoylquinic acid, hederacoside B, C, D, and F, hederaginin-3-O-glucoside, α-, β-, and δ-hederin. Data on their identification and estimated content were published previously [5,15]. The quality of ivy extract is verified by hederacoside C content in compliance with the European Pharmacopoeia. The test item was supplied by Engelhard Arzneimittel (Niederdorfelden, Germany).

### 2.3. Basic Information on In Vivo Studies

Two not-registered study protocols were prepared, with the facility conducting the studies prior to the respective study start. The sample size was decided based on the conducting facility’s previous experience and power analysis. No criteria to exclude data were set for this study, and no experimental unit (=animal) was excluded. To minimize confounders, animals in each cage would receive the same treatment to minimize the impact of any coprophagy. Cages were placed on the IVC racks so that a cage for each treatment would be in the same row (more specifically, details are below for welfare controls). Treatments were administered in a randomized order, one cage at a time. On arrival, animals were randomly placed into cages by the animal husbandry team. They were completely blind to the experimental design. Dosing was conducted blindly by the same individual throughout the study so as to minimize any variation and was based on previous experience in unpublished preclinical studies. Outcome assessments and data analysis were conducted by individuals who were unaware of the treatments given to the animals.

### 2.4. Antitussive Evaluation In Vivo

#### 2.4.1. Animal Housing and Randomization

Thirty-six male Dunkin Hartley guinea pigs (350–500 g, Marshalls BioSource’s Ltd., East Yorkshire, UK) were randomly allocated to a treatment group of six animals each upon arrival based on body weight, resulting in six groups (one vehicle group, one bleomycin + vehicle group, three bleomycin + EA575 treatment groups, one bleomycin + dexamethasone group). Guinea pigs were housed in pens (11,125 cm^2^) with 12 animals each from arrival on, under a 12 h light-dark cycle. Room temperature and humidity were maintained within home office guidelines (17–24 °C and 40–70%, respectively). Environmental enrichment was also provided in all pens. The animals were fed with standard chow (Teklad global diet 2040, Inotiv Inc., West Lafayette, IN, USA), and water was available from bottles, both ad libitum. An acclimatization period of 7 days was allowed before the start of experimental procedures. Body weight was assessed daily.

#### 2.4.2. Bleomycin Challenge and Treatment

Animals were treated by oropharyngeal administration of saline (vehicle group animals only) or bleomycin (BLM, 8 units/kg bw) at a volume of 1 mL/kg bw, under light anesthesia (isoflurane) on Day 1. EA575, dissolved in 4% Tween 80 in PBS, was dosed at 10 mL/kg bw by oral gavage (p.o.) twice a day (BID, every 12 h) each day, starting on Day 1 and concluding on Day 7. Hereby, a dosing regimen of 43, 215.2, and 430.5 mg/kg bw/d was applied. The first dose of EA575 was administered 1 h before the bleomycin challenge. Dexamethasone (3 mg/kg bw, i.p.) was dissolved in PBS and dosed once a day from Day 1 to 7, with the first dose delivered 1 h before the bleomycin challenge.

#### 2.4.3. Tussive Assay and Enhanced Pause Assessment

Seven days after the bleomycin challenge, animals were individually placed into a purpose-built exposure chamber (DSI, New Brighton, MN, USA) with a supplied airflow through a nebuliser of 2 L/min and allowed to settle into their new environment for a 10 min period. Each animal was then exposed for 10 min to an aerosol of 0.2 M citric acid using an ultrasonic nebuliser (Aerogen, Galway, Ireland) set at a nominal liquid consumption rate of 0.6 mL/min. Coughs elicited during the 10 min exposure to the aerosol of 0.2 M citric acid and a subsequent 5 min observation period were recorded using Halcyon Pneumotachs (DSI, New Brighton, MN, USA) to measure pressure changes in a whole-body plethysmography system (DSI, New Brighton, MN, USA), and were also manually counted and analyzed for cough numbers and frequency. Changes in enhanced pause (penH) derived from inspiratory and expiratory flow and time between inspirations were recorded using the whole-body plethysmography system (DSI, New Brighton, MN, USA) and Finepointe software v2.4.6 (DSI, New Brighton, MN, USA).

#### 2.4.4. Bronchoalveolar Lavage—Cell Counts

Immediately after the tussive assessment, the animals were euthanized with pentobarbitone, after which the trachea was isolated by a midline incision in the neck and separation of the muscle layers. A small incision was made into the trachea, and a plastic cannula was inserted and secured in place with a suture. The airways were then lavaged by aspirating the lungs via the tracheal cannula using 5 mL of PBS. The BALF cells were then analyzed for total and differential numbers using an XT-2000iV analyzer (Sysmex Corporation, Kobe, Japan). Cell types classified differentially were neutrophils, eosinophils, lymphocytes, or macrophages.

### 2.5. Mucoactivity Assessment In Vivo

#### 2.5.1. Animal Housing and Randomization

Male C57b6 mice (25–30 g, Charles River UK Ltd., Margate, UK) were housed for 7 days prior to the commencement of the study in cages of 5 and were subjected to a 12:12 h light-dark cycle, with room temperature maintained between 20 and 23 °C and humidity between 49 and 53%. The mice were randomly divided into seven groups of eight animals each based on their body weight (one control group, three EA575 treatment groups, two salbutamol treatment groups, and one EA575 + salbutamol treatment group). Mice were fed a standard mouse chow (RM1 (P) for rodents, SDS, Rosenberg, Germany), and water was available ad libitum. Environmental enrichment was also provided in all cages.

#### 2.5.2. Treatment

EA575, dissolved in 4% Tween 80 in PBS, was administered by oral gavage (10 mL/kg bw) in three dose groups—43, 215.2, and 430.5 mg/kg bw/d. The first dose was administered on Day 1, with dosing occurring twice a day (BID, every 12 h), and the final dose delivered on Day 7, 30 min prior to phenol red administration. Animals receiving the vehicle were also dosed by oral gavage (10 mL/kg bw/d) with a directly comparable BID dosing regimen from Days 1 to 7. Salbutamol administration of 2 or 4 mg/kg bw was applied by oral gavage (10 mL/kg bw) 30 min prior to phenol red administration. For co-administration of EA575 and salbutamol, 430.5 mg/kg bw/d of EA575 and 2 mg/kg bw were applied to one group according to the respective dosing regimen above. Phenol red (1.25% *w*/*v*) was administered intraperitoneally at a dose of 300 mg/kg bw. Thirty minutes subsequently to administration, mice were euthanized by pentobarbitone overdose, and BALF was collected.

#### 2.5.3. Determination of Phenol Red in BALF

For the BALF collection, the trachea was isolated by a midline incision in the neck and separated from the muscle layers. A small incision was made in the trachea, and a plastic cannula was inserted and secured in place with sutures. The airway was then lavaged by flushing out the lungs using 0.5 mL of PBS. This procedure was repeated until the recovered volume was 1.6 mL. BALF was then centrifuged at 2500 rpm for 10 min, from which 500 μL of the resulting supernatant was aliquoted. A total of 50 μL of NaOH (0.1 M) solution was added to the BALF supernatant aliquot, and the absorbance was then measured at 565 nm wavelength using a spectrophotometer. Data are presented as the mean concentration of phenol red (μg/mL) and absorbance units (AU). This procedure was validated by [16] as a method for the identification of mucoactive agents.

### 2.6. Data Analysis

Inter-group deviations were statistically analyzed by a one-way analysis of variance (ANOVA). In cases of significant differences in the mean values among the different levels of treatment, comparisons to the respective group of interest will be carried out using Dunnett’s test. A single animal was considered an experimental unit. *p* < 0.05 was considered statistically significant. Data is shown as mean ± SD. Visualization and statistics were performed using GraphPad Prism v10.2.1.

## 3. Results

### 3.1. EA575 Reduces Cough Events in Acute Lung Inflammation Model

**Figure 1:**
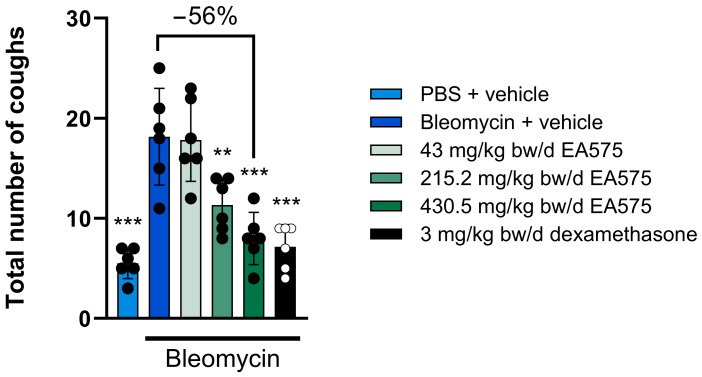
EA575 decreases cough events after citric acid exposure of bleomycin-challenged guinea pigs. The animals (n = 6 per group) were challenged once with phosphate-buffered saline (PBS) or bleomycin (BLM, 2.5 U/kg bw) and treated twice daily for seven days with vehicle, EA575, or dexamethasone. Cough events were recorded in a whole-body plethysmography system for 15 min starting with citric acid exposure. Comparisons between the BLM-only treated animals were made against each of the other groups using a one-way analysis of variance (ANOVA) followed by Dunnett’s test. * *p* ≤ 0.05, ** *p* ≤ 0.01. and *** *p* ≤ 0.001. bw: body weight.

As EA575 is used in the treatment of acute bronchitis, a disease characterized by acute lung inflammation, antitussive effects of EA575 were assessed in an acute lung inflammation model. Induction of acute lung inflammation by bleomycin administration resulted in an increase in cough events within 15 min compared to untreated animals (Figure 1). In detail, 5.5 cough events were recorded in untreated animals versus 18.2 in bleomycin-challenged guinea pigs. The seven-day treatment with EA575 elicited a dose-dependent decrease in citric acid-induced cough events in bleomycin-challenged guinea pigs, with a maximum reduction of up to 56%. Furthermore, in the EA575 high-dose group (430.5 mg/kg bw/d), the cough frequency of 8 coughs in 15 min was comparable to the applied anti-inflammatory positive control of dexamethasone (7.2 cough events in 15 min). These observations indicate a connection between inflammation and cough, suggesting an amelioration of coughing by the anti-inflammatory properties of an agent.

### 3.2. EA575 Alleviates Inflammatory Cell Influx in BALF of an Acute Lung Inflammation Model

**Figure 2:**
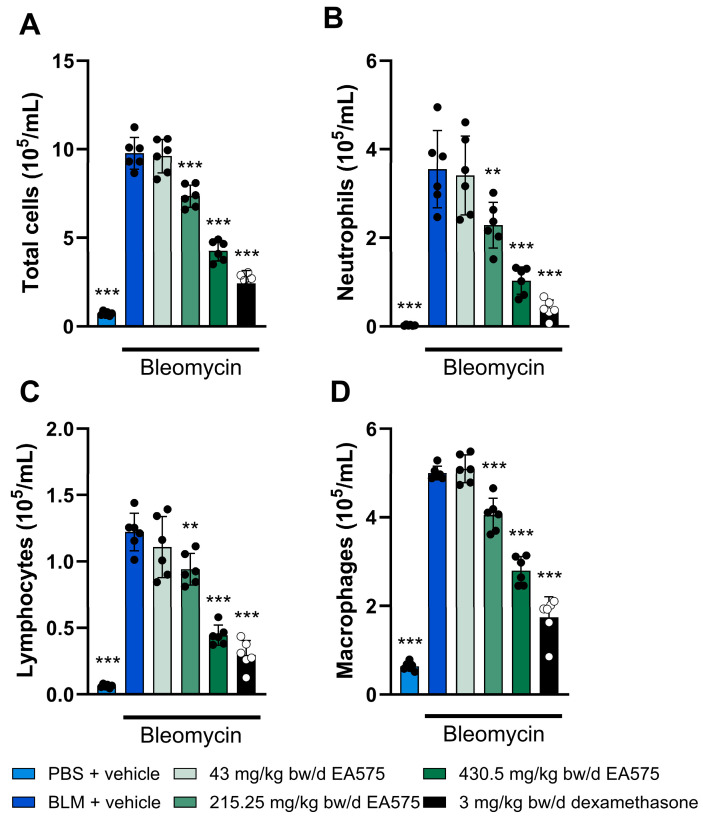
EA575 decreases inflammatory cell influx in the BALF of bleomycin-challenged guinea pigs. The animals (n = 6 per group) were challenged once with phosphate-buffered saline (PBS) or bleomycin (BLM, 2.5 U/kg bw) and treated twice daily for seven days with vehicle, EA575, or dexamethasone. BALF was collected on Day 7 after the BLM challenge in all groups. Data for (**A**) total cell count, (**B**) neutrophils, (**C**) lymphocytes, (**D**) macrophages in BALF are shown as mean ± SD. Comparisons between the BLM-only treated animals were made against each of the other groups using a one-way analysis of variance (ANOVA), followed by Dunnett’s test. * *p* ≤ 0.05, ** *p* ≤ 0.01. and *** *p* ≤ 0.001. bw: body weight.

**Figure 3:**
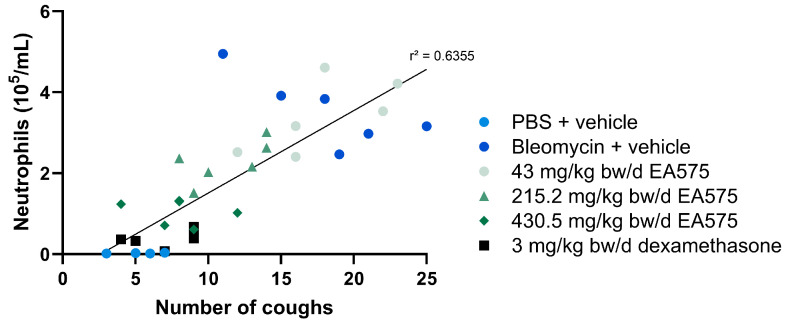
Correlation of cough events and neutrophil count for control and treated guinea pigs. Neutrophil count in BALF was chosen as a representative inflammation parameter and depicted against the number of cough events. A linear regression fit was performed to elucidate the correlation between these observations. bw: body weight.

The administration of bleomycin to guinea pigs resulted in a pronounced inflammatory response, shown by an inflammatory cell influx in BALF (Figure 2). Total cell count, as well as the absolute concentration of neutrophils, lymphocytes, and macrophages, was significantly elevated 7 days post-bleomycin challenge. Following the EA575 treatment, a dose-dependent decrease in the respective parameters was apparent. Hereby, statistical significance was shown within the mid- (215.25 mg/kg bw/d) and high-dose (430.5 mg/kg bw/d) groups for all cell types and the total cell count. In more detail, a maximum reduction of up to 56, 71, 64, and 44% in regard to total cell count, neutrophils, lymphocytes, and macrophages, respectively, was observed. The applied anti-inflammatory positive control (dexamethasone) resulted in a reduction in all BALF parameters investigated. Taken together, this suggests an anti-inflammatory effect of EA575. To further elucidate the correlation between anti-inflammatory effects and the reduction in cough events, these parameters were matched using neutrophil count in BALF as a representative inflammatory parameter, and a linear regression was fitted (Figure 3). The linear regression fit resulted in a correlation coefficient (r) of 0.797 and a coefficient of determination (r^2^) of 0.6355, suggesting a correlation between the reduction in neutrophils in BALF and the number of cough events.

### 3.3. EA575 Attenuates Enhanced Pause in Acute Lung Inflammation Model

**Figure 4:**
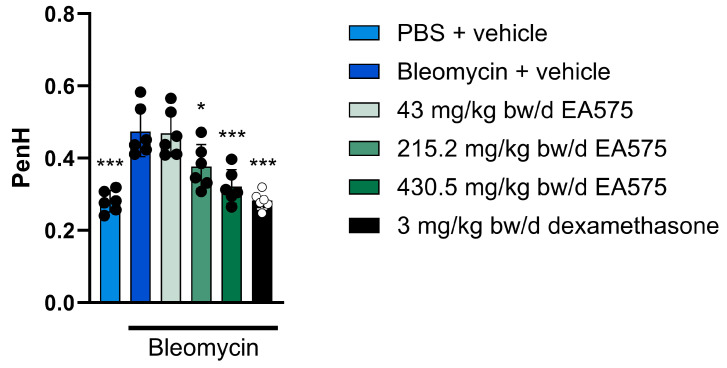
EA575 attenuates enhanced pause increase after bleomycin administration in guinea pigs. The animals (n = 6 per group) were challenged once with phosphate-buffered saline (PBS) or bleomycin (BLM, 2.5 U/kg bw) and treated twice daily for seven days with vehicle, EA575, or dexamethasone. Inspiratory and expiratory flow, as well as the time between inspirations, were recorded using the whole-body plethysmography system. Comparisons between the BLM + vehicle-treated animals were made against each of the other groups using a one-way analysis of variance (ANOVA), followed by Dunnett’s test. * *p* ≤ 0.05, ** *p* ≤ 0.01. and *** *p* ≤ 0.001. bw: body weight.

The treatment with bleomycin resulted in an increase in the enhanced pause value (Figure 4) from 0.28 (PBS-treated control) to 0.47 (bleomycin-treated group). This increase was statistically significant and dose-dependently reduced by the EA575 treatment to 0.38 (215.25 mg/kg bw/d) and 0.32 (430.5 mg/kg bw/d). The treatment with the anti-inflammatory positive control dexamethasone resulted in an enhanced pause value of 0.28, comparable to the PBS-treated control. These observations indicate that inflammation induces airflow impairment through a bleomycin challenge, which is reduced by treatment with anti-inflammatory EA575.

### 3.4. EA575 Increases Phenol Red Secretion in BALF

**Figure 5:**
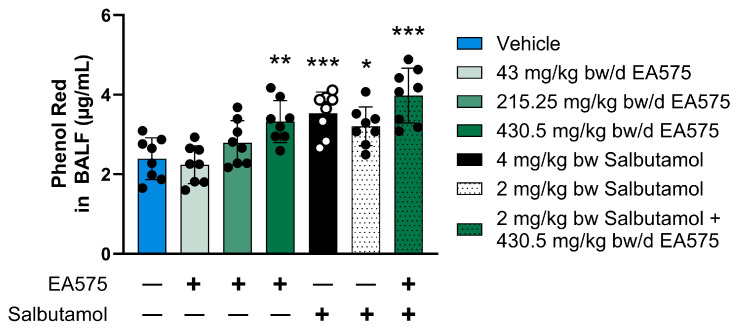
EA575 induces phenol red secretion in BALF and enhances the effect of β_2_ receptor agonist salbutamol. The animals (n = 8 per group) were treated twice daily for seven days with either vehicle or EA575. Treatment with salbutamol at 2 or 4 mg/kg bw occurred once, 30 min prior to phenol red administration. Phenol red (300 mg/kg bw) was applied intraperitoneally 30 min before the study. BALF was collected after study termination. Phenol red concentration was determined in alkaline BALF solution at 565 nm. Comparisons between the vehicle-treated animals were made against each of the other groups using a one-way analysis of variance (ANOVA), followed by Dunnett’s test. * *p* ≤ 0.05, ** *p* ≤ 0.01. and *** *p* ≤ 0.001. bw: body weight.

The 7-day treatment with EA575 resulted in a dose-dependent increase in phenol red concentration in BALF of up to a maximum of 39%, indicating mucoactivity of EA575 (Figure 5). The maximum increase was comparable to the application of 4 mg/kg bw salbutamol (47%), which was used as a positive control. Regarding salbutamol treatment, an increase in phenol red concentration between 2 and 4 mg/kg bw was apparent. The co-administration of EA575 (430.5 mg/kg bw/d, 7 days) and salbutamol (2 mg/kg bw, administered once) resulted in pronounced phenol red concentration compared to the sole treatment of 430.5 mg/kg bw/d EA575 or 2 mg/kg bw salbutamol, respectively. Despite not being statistically significant, this still indicates the synergistic activity of EA575 and salbutamol.

## 4. Discussion

The objective of this study was to elucidate the antitussive effect of EA575, potentially resulting from its anti-inflammatory properties, and to demonstrate its mucoactivity in vivo. To assess the antitussive effect, an acute airway inflammation model of bleomycin-treated guinea pigs was exposed to citric acid, and cough events were measured. It is notable that the assessment of citric acid-induced cough in conscious guinea pigs is regarded as a very valuable model system due to a similar reactivity of the relevant human and guinea pig nervous systems, resulting in a high translational potential [17]. In this study, the animals were treated twice daily with EA575 for 7 days, starting on the day of bleomycin administration. The applied disease model is regarded as a well-established model of idiopathic pulmonary fibrosis. However, during the initial phase (1–7 days), acute lung injury and inflammation occur before drifting into active (Day 7–14) and late (Day 21–28) fibrosis [18,19]. Therefore, studying termination within the early phase after bleomycin administration (1–7 days) allows for the investigation of acute airway inflammation. This can be seen through increased neutrophilia and cytokine secretion, as described in rats and mice after bleomycin treatment [20,21,22]. Neutrophilia was also observed in the course of BALF analysis within this study. The impact of bleomycin treatment on guinea pigs regarding the induction of cough after chemical challenge was shown by Guan and colleagues, demonstrating a clear induction of cough events after capsaicin exposure on Days 7, 14, 21, and 28 following bleomycin challenge [23]. Furthermore, Guo and colleagues investigated capsaicin-induced cough events in guinea pigs on Days 13 and 27 after bleomycin treatment, observing the same effect of increased number of coughs [24]. Both publications suggested that the increased expression of TRPA1 and TRPV1 channels, as well as neurogenic inflammation, may be possible causes of the bleomycin-induced increase in coughing. Besides neurogenic inflammation, virus-induced airway inflammation and the accompanying release of cytokines, such as IFN-γ, TNF-α, or IL-1β, are known to demonstrate an impact on airway hypersensitivity and, therefore, an increase in coughing [25]. For instance, intratracheal instillation of IFN-γ increased cough events in guinea pigs after citric acid exposure [26]. IFN-γ can be secreted by different types of lymphocytes, a cell population that was decreased in a dose-dependent manner by EA575 treatment within this study. It is further described that IFN-γ, along with TNF-α, results in membrane depolarization and accordingly triggers action potentials in vagal sensory neurons and subsequently increases cough sensitivity. Since several receptors for cytokines are apparent on vagal sensory neuron terminals, increased cough sensitivity due to secretion of respective cytokines during inflammatory processes in the lung is suggested [27]. Since neutrophils and macrophages play a crucial role in cytokine secretion, it is hypothesized that the observed reduction in inflammatory cell influx in BALF within this study results in reduced cytokine secretion. A EA575-related decrease in secretion of several cytokines, e.g., IFN-γ, TNF-α, and IL-1β, was also observed in a murine acute lung inflammation model (manuscript in preparation). Furthermore, a reduction in IL-12, IL-23, IL-27, IL1-β, IL-6, and TNF-α secretion in immature human monocyte-derived dendritic cells by EA575 was recently described [8] and is in concordance with further in vitro data describing EA575′s anti-inflammatory potential, such as reduced IL-6 secretion or decreased NFκB activation [6,7,9,10]. Taken together, this indicates that the observed reduction in cough events after EA575 treatment in the course of acute airway inflammation is due to its anti-inflammatory effects and not because of acting as a centrally acting cough suppressant. It is tempting to speculate that this hypothesis is the cause of the reduction in cough intensity and attacks identified in a non-interventional study following treatment with the EA575-containing medicinal product [28,29]. The improvement of cough symptoms in accordance with the treatment is further summarized in two reviews [2,3].

Enhanced pause (PenH) represents a non-dimensional parameter that can be assessed in conscious animals as a non-invasive method and is described by the ratio of exhalation to inhalation peak flow rates and the ratio of exhalation cycle duration to 36% of the exhalation cycle. It can be calculated, as previously described [30]. PenH is regarded critically as a parameter of airway function since it is dependent on various exogenous factors, e.g., stress, humidity, or temperature, and should rather be seen as a measure of breathing patterns [31,32]. Therefore, the interpretation of the respective results of this parameter needs to be performed with caution. Nonetheless, an increase in PenH in airway inflammation disease models is apparent, for instance, in murine allergic airway inflammation models [31,33,34], ozone-induced acute lung inflammation in rats [35], or viral-induced inflammation in mice [36,37]. Condor Capcha and colleagues postulated that an increase in PenH after viral infection may be linked to airway inflammation and respective mucus secretions [36]. A connection between acute airway inflammation and an increase in PenH was also seen within the presented data after bleomycin-induced acute lung inflammation. Furthermore, a decrease in PenH after EA575 treatment to nearly the level of untreated healthy animals was observed in this study. This indicates that treatment with EA575 restores conditions in the lung comparable to those of healthy animals, suggesting potentially biological relevance. However, it must be stated that PenH does not represent a reliable parameter for lung function; therefore, this conclusion must be seen as a hint for future research on the effect of EA575 on improving lung function within acute inflammatory conditions. The observation of decreased PenH further correlates with the decrease in inflammatory cells in BALF. Furthermore, a reduction in goblet cell metaplasia by EA575 treatment in a murine bleomycin-induced airway inflammation model was observed (manuscript in preparation). This is suggested to result in less mucus secretion and might further highlight the postulation of Condor Capcha and colleagues.

Representing the first barrier of host defense in the lung, mucus is built up by two layers: the gel layer blocking pathogen penetration and the periciliary liquid layer facilitating ciliary beating and thus mucociliary clearance [38]. Mucus consists of water, high molecular weight glycoproteins, so-called mucins, and a mixture of proteins and lipids. It derives from different origins, namely submucous glands, goblet cells, and epithelial cells [38,39]. In a healthy state, the mucus is secreted in a physiological manner, resulting in clearance by ciliated epithelial cells and subsequent swallowing [40]. However, in diseases such as asthma, chronic bronchitis, and also acute viral-induced airway inflammation, excessive mucus secretion occurs, potentially resulting in airway obstruction [38,40]. Mucus hypersecretion is, therefore, at least partially a consequence of inflammation-related goblet cell and submucosal gland hyperplasia and hypertrophy [38,39,40]. Mucoactive drugs help to alleviate mucus hypersecretion in different ways and are, therefore, classified as expectorants, mucolytics, mucokinetics, and/or mucoregulators [39,40]. Hereby, expectorants are thought to increase mucin production and/or mucus hydration to a point that eases the discharge of mucus by coughing [16,39,40]. Hence, by determining phenol red secretion in BALF after the respective intraperitoneal treatment, the capacity of the substance to act as an expectorant was assessed in this study. Therefore, the increase in phenol red in BALF after EA575 treatment indicates its ability to act as an expectorant via a secretolytic mode of action. The ability of EA575 to act as a mucoactive drug was also identified in various clinical studies, revealing positive effects on expectoration [41,42,43]. The method applied here was validated by Menezes and colleagues, testing various substances with secretolytic properties as positive controls, such as ambroxol, ammonium chloride, and salbutamol [16]. However, the mode of action for these reference substances is diverse and still quite unclear. Ambroxol, for instance, might induce a secretolytic effect by increasing respiratory fluid volume, increasing mucus secretion, and/or enhancing surfactant secretion [44]. Furthermore, it can be hypothesized that salbutamol acts in concordance with salmeterol, as both are known β_2_ receptor agonists. For salmeterol, a decreased ion concentration and increased hydration level of mucus inside cystic fibrosis secretory granules were observed in vitro [45]. In addition, ambroxol and salbutamol are both listed as mucokinetics rather than expectorants [40]. This highlights that mucoactive agents may obtain more than one mode of action in order to exert their effect. Regarding EA575, an additional classification as mucoregulator can be suggested due to its anti-inflammatory effect and amelioration of goblet cell metaplasia in a murine acute airway inflammation model (manuscript in preparation). Furthermore, mucokinetic properties are hypothesized due to possible surfactant secretion based on enhanced cellular cAMP concentration after β_2_ adrenergic receptor activation [14]. Respective receptor activation and increased cellular cAMP levels after EA575 treatment have been shown in vitro [9,11]. All in all, the mucoactivity of EA575 is indicated, but further research is needed to further clarify modes of action. It is further notable that the co-administration of EA575 and salbutamol resulted in an increased phenol red secretion compared to salbutamol treatment alone. It is known that EA575, especially its constituent α-hederin, affects the stimulation of β_2_-adrenergic receptors. In more detail, the mode of action derives from enhanced β_2_-adrenergic responsiveness mediated by inhibition of GRK2-related phosphorylation of activated β_2_-adrenergic receptors. This results in reduced β_2_-adrenergic receptor internalization during respective stimuli [46,47] and, therefore, enhanced efficacy of endogenous or exogenous β_2_ receptor agonists. The presented data on the in vivo co-administration of EA575 and salbutamol provide empirical evidence that validates the postulated in vitro mode of action. Furthermore, it opens space for discussions on the beneficial use of potential co-medication with EA575 in therapies that involve β_2_ receptor agonists, e.g., through the potential use of decreased β_2_ receptor agonist doses for the same effect as β_2_ receptor agonist alone, resulting in fewer side effects. This might further result in a decrease in tachyphylaxis effects, which can be a benefit in clinical uses.

## 5. Conclusions

In conclusion, our data reveals an antitussive effect of EA575 in an in vivo acute airway inflammation model. This effect can be explained by its anti-inflammatory properties rather than acting as a direct cough suppressant. Further, a change towards normalization in the breathing pattern in guinea pigs with acute airway inflammation after EA575 treatment was shown, hypothesizing a relief of adverse inflammation-associated effects such as mucus hypersecretion. Lastly, the ability of EA575 to act as a mucoactive drug is due to a secretolytic effect, at least partially based on the prolonged activation of the β_2_-adrenergic receptor response. Taken together, these effects highlight the benefits of EA575 in the treatment of inflammation-related airway diseases accompanied by cough, such as acute bronchitis. Namely, the reduction in inflammation-associated cough and the easing of the removal of excessive mucus removal by acting as a mucoactive drug. Furthermore, the data might also form the foundation for potential use in chronic inflammation-related airway diseases.

## Data Availability

All available data are reported in this article.

## Abbreviations

The following abbreviations are used in this manuscript:

BALF	Bronchoalveolar Lavage Fluid
BLM	Bleomycin
bw	Body weight
PBS	Phosphate-buffered saline
PRR	Pattern-recognition receptor
RSV	Respiratory syncytial virus
TLR	Toll-like receptor
